# Emotion, rationality, and decision-making: how to link affective and social neuroscience with social theory

**DOI:** 10.3389/fnins.2015.00332

**Published:** 2015-09-22

**Authors:** Marco Verweij, Timothy J. Senior, Juan F. Domínguez D., Robert Turner

**Affiliations:** ^1^Department of Social Sciences and Humanities, Jacobs University BremenBremen, Germany; ^2^Digital Cultures Research Centre, University of the West of EnglandBristol, UK; ^3^Social Neuroscience Lab, School of Psychological Sciences, Monash UniversityMelbourne, VIC, Australia; ^4^Department of Neurophysics, Max Planck Institute for Human Cognitive and Brain SciencesLeipzig, Germany

**Keywords:** affective and social neuroscience, social and political theory, somatic marker hypothesis, plural rationality

## Abstract

In this paper, we argue for a stronger engagement between concepts in affective and social neuroscience on the one hand, and theories from the fields of anthropology, economics, political science, and sociology on the other. Affective and social neuroscience could provide an additional assessment of social theories. We argue that some of the most influential social theories of the last four decades—rational choice theory, behavioral economics, and post-structuralism—contain assumptions that are inconsistent with key findings in affective and social neuroscience. We also show that another approach from the social sciences—plural rationality theory—shows greater compatibility with these findings. We further claim that, in their turn, social theories can strengthen affective and social neuroscience. The former can provide more precise formulations of the social phenomena that neuroscientific models have targeted, can help neuroscientists who build these models become more aware of their social and cultural biases, and can even improve the models themselves. To illustrate, we show how plural rationality theory can be used to further specify and test the somatic marker hypothesis. Thus, we aim to accelerate the much-needed merger of social theories with affective and social neuroscience.

## Introduction

Affective and social neuroscience have rapidly advanced during the last 25 years (Baron-Cohen et al., 2013; Dębiec et al., 2014). As a result, insights generated in these fields have begun to influence theorizing in the social sciences (e.g., Franks, 2010; Vander Valk, 2012). Similarly, social theories have also started to inform affective and social neuroscience (Whitehead, 2001; Domínguez D. et al., 2010), albeit to a lesser extent. In this paper, we build on these initial efforts to link affective and social neuroscience with social theories. We define social theories as conceptual frameworks from anthropology, economics, political science, and sociology that specify how people interact with, and exercise power over, each other. We argue that affective and social neuroscience and social theories can strengthen each other—and show how this can be done.

Affective and social neuroscience can support the social sciences by offering additional assessments of the assumptions that social theories make regarding cognition, emotion, decision-making, and social behavior. It would be problematic if such theories rested on premises that are inconsistent with insights that have been carefully collected in brain research. In turn, frameworks from anthropology, economics, political science, and sociology can help efforts to formulate and specify neuroscientific models. Increasingly, brain researchers have become interested in identifying neuronal networks involved in social interactions (Pfeiffer et al., 2013), political choices (Schreiber et al., 2013), ethical behavior (Domínguez D., 2015), and other social phenomena. Social science approaches can be of use to these efforts by offering reliable and empirically valid definitions of phenomena that affective and social neuroscientists seek to explain. How, for instance, can one hope to uncover the neuronal correlates of social interactions without a solid grasp of the types of social relations people tend to engage? This is the kind of information that social theories can provide. Furthermore, these theories can suggest parts of the neuronal networks that enable human emotion, decision-making, and behavior. That is to say, approaches from anthropology, economics, political science, and sociology can sometimes serve as a source of hypotheses for the independent variables used in neuroscientific models (Vogeley and Roepstorff, 2009). For example, in this paper we show how the somatic marker hypothesis proposed in affective and social neuroscience can be specified more fully with the help of a theory developed in anthropology and political science.

We build our case for a further integration of social theory and brain research as follows. In the first half of the paper, we demonstrate how affective and social neuroscience can enhance theorizing in anthropology, economics, political science, and sociology. We do so by focusing on four types of social theory: rational choice analysis, behavioral economics and public policy, post-structuralism, and plural rationality theory. We show that the first three types of theory contain assumptions about human cognition, emotion and decision-making that are not fully consistent with present understandings of how the human brain functions. We also argue that the fourth type appears more plausible from the viewpoint of affective and social neuroscience. In the second half of the paper, we explore how social theories can contribute to brain research. We combine the plural rationality (or cultural) theory pioneered by anthropologist Douglas with the somatic marker-hypothesis developed by Damasio, so as to form a model of the neuronal networks involved in how humans evaluate social situations. We argue that if this model were empirically valid, then it would not only extend the somatic marker hypothesis, but would also solve a remaining conceptual puzzle of Douglas' theoretical framework. We conclude by discussing how this model can be tested with neuroscientific means.

## How affective and social neuroscience can improve social theory

As we mentioned above, affective and social neuroscience can provide an additional test for social theories (besides the internal coherence of, and empirical evidence for, these theories). We first describe various key findings from affective and social neuroscience with direct relevance for social theorizing. Thereafter, we introduce four general theories that currently abound in anthropology, economics, political science, and sociology, paying particular attention to their treatment of emotions, rationality, and decision-making. Finally, we argue that only one of these appears to be fully consistent with brain research.

### Key insights from affective and social neuroscience

The closely related fields of affective and social neuroscience have thrived in the past few decades. Despite a variety of continuing debates and disagreements, there appears to be (near) consensus on a number of points. Nine of these have direct relevance for theorizing in anthropology, economics, political science, and sociology.

A first point of agreement is that people are deeply concerned with, and influenced by, their social relations. The human brain enables, makes use of, and is partly shaped by a wide array of social interactions (Turner, 2001; Cacioppo and Patrick, 2008; Gazzaniga, 2008; Fouragnan et al., 2013; Lieberman, 2013). This is compatible with the social brain-hypothesis, the leading explanation of the expansion of the human brain during the course of evolution. According to this hypothesis (Dunbar and Shultz, 2007), among primates, the size of the neocortex (as compared to the whole brain) correlates with many indices of social complexity, including social group size, grooming clique size, the frequency of coalitions, male mating strategies, the prevalence of social play, the rate of tactical deception, and the frequency of social learning. Hence, we are “wired to be social” (Castiello et al., 2010).

Second, affective and social neuroscience have particularly focused on two aspects of social relations, namely, social dominance (Zink et al., 2008; Chiao et al., 2009b; Ray et al., 2010; Mason et al., 2014) and social identification (Chiao et al., 2009a; Kitayama and Park, 2010; Cikara et al., 2011; Amodio, 2014). Social dominance entails the establishment of status differentiation among people. Social identification stands for the formation of group boundaries, which turns a singular “I” perspective into a plural “we” perspective, while at the same time creating distinctions between “us” and “them.” The processing, in the human brain, of these two elements of social interaction influences neuronal networks involved in attention, perception, evaluation, memory, and emotion (Cikara and Van Bavel, 2014).

Third, the neuronal networks that facilitate social interaction and decision-making are likely to have enabled other cognitive functions as well (Frith, 2007a). Although the primary evolutionary driver of human inventiveness appears to have been increasing social complexity, this creativity, once it had emerged, could be used for many other purposes as well. In Damasio's words (1994), “It is plausible that a system geared to produce markers and signposts to guide ‘personal’ and ‘social’ responses would have been co-opted to assist with ‘other’ decision-making. The machinery that helps you decide whom to befriend would also help you design a house in which the basement will not flood.”

Fourth, emotions, and especially social emotions (such as empathy, admiration, spite, and jealousy), are pivotal to social decision-making (LeDoux, 1998; Panksepp, 1998). An emotion can be defined as “the process by which the brain determines or computes the value of a stimulus” (LeDoux, 2002). Emotions do not necessarily determine our social choices, and can even be deliberately reappraised (Ochsner et al., 2002), but at a minimum they limit and bias our decisions. Neurologically impaired patients, who display flat emotions, often find it hard to take personally beneficial, and socially appropriate, decisions (Damasio, 2005).

Fifth, emotions can be distinguished from feelings (or affects). The latter are the consequence of emotions sufficiently intense to be noted consciously (Damasio, 1999; Panksepp, 2005; LeDoux, 2008). From this follows that not every time a person has a social emotion, it will reach his or her awareness (i.e., give rise to a feeling). As a result, social decision-making is to a significant degree non-deliberate (Purves, 2010).

Sixth, although social emotions can in principle be separated from social cognition, in practice they are highly intertwined and mutually dependent (Phelps et al., 2014; Inzlicht et al., 2015). After a comprehensive review of current empirical evidence, Pessoa (2013) concludes that “labels such as ‘perception,’ ‘cognition,’ and ‘emotion’ are useful linguistic categories, but only in the limited sense of providing placeholders for descriptive purposes – they do *not* map onto behavior *or* the brain.”

Seventh, emotions and feelings have deep evolutionary roots, and serve the organism's survival by ensuring homeostatic processes (Craig, 2002; Panksepp and Biven, 2012; Damasio and Carvalho, 2013). The neuronal circuits involved in the processing of emotions are conserved throughout mammalian evolution, and are therefore present in humans as well as other mammals (LeDoux, 2002). This implies that subcortical nuclei (including the amygdala, hypothalamus, hippocampus, and brainstem nuclei), and evolutionary older parts of the cortex (such as the insula), have central roles in the formation of emotions, feelings, and social decision-making (Parvizi, 2009). Subcortical areas are more primitive than cortical regions, and therefore vary much less between members of the same species and even across species (Gazzaniga et al., 2002). This does not mean that ancient parts of the brain dictate emotions, feelings, and social decision-making. As Pessoa (2013) emphasizes, in the course of evolution, older brain regions, such as the amygdala and hypothalamus, have been fully embedded into new neuronal networks, so as to generate novel functions and support richer patterns of interaction with physical and social environments. As a result, subcortical and cortical areas are highly integrated. Nevertheless, in this process, each prior form of neuronal organization has severely constrained and shaped the emergence of new capabilities (Anderson, 2015). Hence, Edelman (2006) maintains that we have a “thalamocortical brain,” while Damasio (2010) writes of the “anatomical bottleneck behind the conscious mind,” which is responsible for “the sameness that hallmarks the repertoire of human behavior.”

Eighth, although brain researchers have proposed diverse lists of emotions, most of them acknowledge that emotions can be assembled into two categories: negative (or aversive) and positive (or appetitive) ones (Damasio, 1999; Panksepp, 2010; Kandel, 2012). The former include for example, fear, disgust, and embarrassment, while the latter encompass trust, love, and admiration. The reason for this dichotomy appears to be that, in evolutionary terms, emotions have been derived from the neuronal and bodily networks involved in withdrawal or approach reactions (Adolphs, 2002; Gazzaniga, 2008).

Last, an important manner in which emotions promote survival is by facilitating learning and memory (LeDoux, 2002; Immordino-Yang and Damasio, 2007). Emotions can be understood as reflecting, and summing up, an organism's experiences with similar social and other situations in the past. As a consequence, it appears likely that cultural influences on people's behavior and thought function, at least partially, through their impact on social emotions and feelings (Immordino-Yang, 2013).

These nine insights generated in affective and social neuroscience have important implications for theorizing in anthropology, economics, political science, and sociology. As we argue in the next section, various—but not all—social theories make assumptions that are inconsistent with these insights. In view of the multitudes of social theories that have been formulated, it is not feasible to discuss all approaches. We therefore limit ourselves to four theoretical camps that are currently particularly influential across the social sciences: rational choice analysis, behavioral economics and public policy, post-structuralism, and plural rationality theory.

### Rational choice analysis

The first versions of rational choice theory were formulated in the 1960s and 1970s (Riker, 1962; Olson, 1965; Niskanen, 1971). It entailed the extension of interbellum neoclassical economics to the fields of sociology, political science, and anthropology (Becker, 1976; Laitin, 1986; Coleman, 1990; Scharpf, 1997). By the 1990s, rational choice theory had established itself as the dominant approach in political science and (under the label of expected utility theory) in economics, and had also become influential in sociology and, to a far lesser extent, anthropology (Hindmoor, 2006).

According to rational choice analysis, actors are highly informed about the options available to them, can consistently and uniquely rank all options according to their preferences and, based on these calculations, choose the most satisfactory option—while also taking into account the choices that other actors are likely to arrive at. In other words, the theory assumes that actors' choices are the outcomes of an exhaustive cost-benefit analysis of all the courses of action that are available to them, as well as to other actors. Emotions play little role in this decision-making process. In neglecting the role of emotions, rational choice theory is out of step with present-day neuroscience—a point that has been made by a variety of brain researchers (e.g., Damasio, 1994; Koenigs et al., 2007; Krajbich et al., 2009). In addition, affective and social neuroscience are at variance with rational choice analysis' under-socialized depiction of humans. In rational choice theory, an individual's choices are only strategically affected by those of others. In other words, people's preferences are not supposed to be influenced by the social structures in which they partake. This is inconsistent with the highly social nature of the brain.

Still, some scholars have sought to defend rational choice analysis with the help of neuroscience (Glimcher et al., 2005; Padoa-Schioppa and Assad, 2008). They have done so by analyzing how the brain computes and compares the values of different choices before reaching a final decision. In and of itself, this is important research as it provides further insight into the neuronal circuits for processing decisions. However, these scholars have also asserted that their findings underpin expected utility theory/rational choice analysis. This may be the case, but many social theories, including the ones discussed in this paper, presume that actors compare the appeal of different options before reaching a decision. Various approaches, and not just rational choice theory, therefore appear to be compatible with these findings.

### Behavorial economics and public policy

The neuroscientific critique of rational choice theory's premises coincided with accumulating empirical evidence from the social sciences that its predictions were frequently inaccurate as well (e.g., Green and Shapiro, 1994; Henrich et al., 2001). As a result, social scientists began to reconsider the roles that emotions play in human life. Many have done so by building on dual-systems (or dual process) models developed in psychology. These models distinguish between two modes of thinking and deciding (Chaiken and Trope, 1999; Stanovich and West, 2000; Kahneman, 2003). In the first of these two modes, usually dubbed System 1, thinking and deciding are fast, automatic, intuitive, effortless, slow-learning, and emotional. By contrast, in System 2, thinking and deciding are slow, deliberate, effortful, rule-governed, emotionally neutral, as well as relatively adaptable. Often, it is assumed that these modes correspond to distinct neuronal networks in the brain, one of which (System 1) is evolutionary older than the other. According to dual-systems models, human cognition and decision-making are routinely processed in System 1. Although less precise, this mode allows people to choose satisfactory courses of action more often than not. It is occasionally overridden and corrected by System 2, which is slower and demands more effort, but is also more accurate. The consequence of people's preponderant reliance on System 1 is that although human decision-making is often adequate for life's purposes, it still displays systematic biases and fallacies. These errors explain how and why humans fail to display the behavior predicted by rational choice analysis and sometimes embark on courses of action that go against their own interests.

Beyond psychology, dual-systems models have been used in other disciplines to develop such approaches as behavioral economics (Thaler, 1993; Smith, 2005), behavioral law (Sunstein, 2000), and behavioral public policy (Oliver, 2013). These approaches employ the biases and fallacies highlighted by dual-systems models to explain a set of seemingly irrational behaviors in finance, consumption, voting, law abidance, *etc*. Behavioral theories have become quite influential in and beyond academia during the last two decades. Chairs, graduate programs, academic societies, annual conferences, and handbooks have sprung up, thus institutionalizing these approaches. In recent years, the US and UK governments, as well as the European Commission, have used behavioral insight teams to inform future policy-making (Oullier, 2013). Last, behavioral economics has had a significant influence on the development of neuroeconomics (Camerer et al., 2004). Not all neuroeconomists are behavioral economists, but most of them are (Ross, 2008). Much effort in neuroeconomics has therefore gone into attempts to uncover the distinct neuronal networks subserving the emotional/intuitive and cognitive/deliberative modes of decision-making (e.g., McClure et al., 2007; Albrecht et al., 2010).

Even though behavioral approaches have risen to prominence in the social sciences, the main premise on which they are built—namely, that emotion and cognition can be treated as separate—is incongruent with neuroscientific evidence showing that these two functions are largely integrated in the brain and are mutually enabling. It is therefore not surprising that attempts to pinpoint the neuronal substrates of Systems 1 and 2 have had contradictory results. Specific brain regions have been associated with intuitive judgments in one study and with deliberate judgments in another (Volz and von Cramon, 2008). Neuroscientific criticisms of the efforts to create more realistic models of human decision-making by adding a category of apparently irrational emotions to supposedly more rational cognition have been joined by other objections. Volz and Gigerenzer (2014) have argued that lack of conceptual precision has rendered dual-systems models, behavioral approaches, and neuroeconomics hard to falsify. Moreover, scholars have criticized the psychological experiments that have purportedly shown the existence of biases and errors in human decision-making (Jussim, 2012). For example, Stein (2013) has argued that the choices made in these experiments can easily be interpreted as rational rather than fallacious. This can be done by judging them against the standard of Baconian (as opposed to Bayesian) probability.

### Post-structuralism

Another influential camp in the social sciences during the last 40 years has been post-structuralism. During this period, post-structuralism has become the hegemonic perspective in social anthropology, has dominated subfields of sociology (such as the study of science, technology, and society), and has become quite influential as well in political science. It first emerged in French anthropology and philosophy in the 1960s and 1970s (Foucault, 1970; Derrida, 1976; Barthes, 1982; Deleuze, 1995), where it attempted to provide the ongoing student revolts with a theoretical footing. Soon, it spread to other parts of the world (e.g., Rorty, 1979; Spivak, 1988; Žižek, 1989).

Post-structuralism posits that generalizations about social and political life are not possible—at least not without overlooking vital parts of empirical reality or promising avenues for social change. True to form, many post-structuralists also resist generalizations of their own writings, and sometimes reject the label itself. Nevertheless, many people agree that a substantive number of publications in the social sciences contain the following features and can be usefully grouped under the banner of post-structuralism (Belsey, 2002). According to the approach, human social and emotional life is highly malleable. That is to say, post-structuralism denies the existence of constraints on how people can organize, perceive, justify, and emotionally experience their social relations. This conclusion follows from the post-structuralist take on language. Post-structuralism holds that language intervenes between individuals on the one hand, and external reality on the other. Humans cannot perceive anything but through language. It shapes the very categories with which we think, and thus creates social reality. Moreover, language is built from arbitrary collective constructions (often in the form of binary opposites—such as man/woman, North/South, white/black, emotional/rational). It is possible to think about these collective constructs, but we can only do so with the help of language. Hence, we are then still relying on the assumptions and differences buried deep within language. It is therefore not possible to escape the web of language; we are forever trapped in it, and do not have direct access to outside reality. This inability to achieve objectivity means that it is not possible to generate universally valid theories of social and emotional life. Moreover, the primacy and arbitrariness of language and discourse ensure that people's social and emotional lives are highly fluid. In the words of Tamboukou (2003), Foucault and Deleuze “would both refuse to produce a generic theory about emotions, since they refuse any universal or primordial notion of the human essence as such…. [A]ffects follow ‘lines of flight,’ escaping planes of consistency and following unpredictable directions.” All that is therefore left for social analysis is to reveal the power positions privileged by, and the arbitrariness and contradictions within, existing discourses.

Post-structuralists were quick off the mark in claiming that neuroscience validates their approach (Connolly, 2002; Turner, 2002). They have argued that because a person's brain displays plasticity, is wired in unique ways, and makes use of non-rational and non-conscious emotions, human social life must be highly malleable. But this conclusion is arrived at by a leap across logical gaps. It is empirically valid to state that the human brain exhibits changes in the connective strength between neurons, is shaped by social and cultural relations, and that no two brains are the same. However, such changes and differences are by no means boundless. Not only is the general architecture of the human brain remarkably similar across individuals (Dehaene, 2009), but the “what, where, when, why, and how” of neuronal plasticity must be understood as operating within strict limits. Our current understanding of neuronal plasticity may only be rudimentary, but it is clear that the plastic capabilities of the brain are necessarily constrained to preserve core, overall functionalities (Gazzaniga, 2008). Second, in positing that emotions have an independent influence on social interactions and decision-making apart from reasoning (Ross, 2006; Gregg and Seigworth, 2010; Braunmühl, 2012), post-structuralists—like behavioral economists—overlook the abundant empirical evidence showing that emotions and cognition are highly intertwined. Last, by insisting that social and emotional life is plastic, post-structuralists ignore the major roles that subcortical nuclei play in the formation of emotions, feelings, and decisions. In particular, the amygdala performs a central part in decision-making by matching different emotions to types of social situations (LeDoux, 2000). The crucial roles that subcortical parts fulfill in human decision-making imply that the social and emotional life of humans (and other animals) is subject to severe constraints. In sum, current affective and social neuroscience does not accord with post-structuralist premises about emotions, language, and social life (Frith, 2007b).

### Plural rationality theory

A final camp in the social sciences that we discuss consists of plural rationality theory. This approach was first developed by anthropologist Douglas (1970, 1978, 1982), and then extended to economics (Douglas and Isherwood, 1996), political science (Hood, 1998; Wildavsky, 2005; Swedlow, 2014), and sociology (Thompson et al., 1990; Rayner, 1992; Thompson, 2008). In earlier applications, the approach was usually called “cultural theory,” but to avoid confusion with post-structuralist cultural analyses, the term “plural rationality theory” is often preferred nowadays.

This framework states that any social domain consists of dynamic combinations and reinventions of four ways of life, namely hierarchy, egalitarianism, individualism, and fatalism. Each way of life comprises a particular pattern of organizing social relations, and a corresponding cultural bias. The latter includes perceptions, beliefs, and emotions (Perri 6, 2002). According to the theory, these patterns of social relations and cultural biases are mutually supportive: engaging in a certain pattern of social interaction induces people to adopt a particular cultural bias and, *vice versa*, adopting a cultural bias encourages people to interact in a certain manner. As each way of life is different from, but also co-dependent on, the other ways of life, any social domain contains traces of all four patterns of social relations and their corresponding cognitive and emotional biases. In plural rationality theory, these four ways of life result from assigning “high” and “low” values to two underlying social dimensions, namely grid (or stratification; the extent to which social roles are ranked and differentiated) and group (or collectivity; the extent to which people feel part of a larger social whole beyond the individual). Four alternative ways of organizing, perceiving, and experiencing thus emerge: hierarchy (high grid, high group); egalitarianism (low grid, high group); individualism (low grid, low group); and fatalism (high grid, low group). Although people's emotions and cognition are assumed to be influenced by the social relations in which they engage, they retain agency and rationality. Individuals compare the truth-claims (about nature, human nature, time, space, *etc*.) of their cultural biases with their perceptions of reality. If the discrepancy between their expectations and perceptions becomes too vast, people will eventually opt for another way of life. People's choices for particular ways of life are therefore based on the accumulation of experiences, i.e., on learning. Moreover, the theory recognizes that an individual can adhere to alternative ways of life in different social contexts.

Plural rationality theory appears compatible with the insights into decision-making and behavior provided by affective and social neuroscience (Turner and Whitehead, 2008). To start, the theory assumes that humans are inherently social. Not just in the shallow sense that individuals tend to engage in many social relations, but also in the deeper meaning that people's cognition and emotions are influenced by the social structures in which they live. In addition, plural rationality theory does not separate cognition, emotion and social relations. Each of its ways of life includes perceptions (of human nature, risk, time, space, justice, *etc*.), cognition (such as information-processing styles), emotional likes and dislikes, as well as a pattern of social relations (Douglas and Ney, 1998). Furthermore, in accordance with neuroscience, the theory assumes that people's preferences for patterns of social relations enable their other, ostensibly non-social choices. As Douglas (1996) puts it, “When we choose a doctor, or a wallpaper design, or china and cutlery for the table, and especially when we eat and when we decide not to eat meat, the choice is part of a pattern of choices that we are making with friends.”

Plural rationality theory also recognizes that human behavior and decision-making are constrained. It acknowledges that social and cultural life is highly diverse across time and space. Yet it states that this diversity is at least partially the outcome of the waxing and waning, as well as merging and splitting, of just four ways of organizing, perceiving, and experiencing social relations in every social domain. This combinatorial principle makes sense in light of the constraints on human emotion, cognition, and decision-making that are the consequence of the central roles played in the brain by subcortical nuclei. Indeed, this principle has frequently been proposed in neuroscience (Jackendoff, 2007; Cacioppo and Patrick, 2008; Changeux, 2008). As Dehaene (2009) puts it, “the human capacity for invention is not endless but is narrowed by our limited neuronal construction set. If human cultures present an appearance of teeming diversity, it is because an exponential number of cultural forms can arise from the multiple combinations of a restricted selection of fundamental cultural traits.” As the evolution of the human brain has involved the embedding of older brain regions (such as the amygdala and hypothalamus) into new neural networks so as to generate novel functions and richer interactions with physical and social environments, limits must exist on the number of ways in which people can organize, perceive, and experience social relations (Anderson, 2015). The existence of such limits is plural rationality theory's core assumption.

Moreover, the constraints that are highlighted by the approach are rooted in two social dimensions that have been found, within social neuroscience and social psychology, to significantly impact attention, perception, evaluation, memory, and emotion (Cikara and Van Bavel, 2014). Douglas' grid-notion corresponds closely to what in these other fields has been conceptualized as social dominance (e.g., Beasley et al., 2012). Her group-concept resembles the manner in which social identification has usually been operationalized, namely as the distinction between interdependence (or collectivism) and independence (or individualism) (Markus and Kitayama, 1991; Han and Northoff, 2008). Finally, in plural rationality theory, people's emotional and cognitive preferences for particular ways of organizing are the outcomes of accumulated experiences with interactions in social and physical environments. As a consequence, they tend to change only after major surprises. In other words, the approach posits that people's emotional and cognitive biases are sticky, and will often only change after an encounter, or set of encounters, that defies established assumptions and expectations. This is in line with current insights in brain research (Bechara and Damasio, 2005). If a person's emotions in a particular social situation reflect, and sum up, his or her experiences in similar, previous situations, then these emotions can be expected to significantly shift only after the occurrence of an important, unexpected event within that type of social situation.

In conclusion, insights developed in affective and social neuroscience appear to be inconsistent with key premises of rational choice analysis, behavioral economics, and public policy, as well as post-structuralism, while they appear to be in line with the assumptions of plural rationality theory. We are not suggesting that the latter is the only social theory that may be consistent with affective and social neuroscience. Others may include the relational models theory developed by Fiske (Fiske, 1991; Jackendoff, 2007), and the heuristics program established by Gigerenzer and colleagues (Volz et al., 2006; Hertwig et al., 2013). It stands to reason to focus more on such approaches when merging the social sciences with brain research.

## How social theory can improve affective and social neuroscience

In the introduction, we listed several contributions that the social sciences can—and should—make to the further development of brain research. These include the provision of: (1) theoretically rigorous, and empirically endorsed, formulations of the types of human behavior and concerns that neuroscience has begun to investigate (such as trust, justice or beauty); and (2) suggestions as to which neuronal networks may be involved in the brain processing of certain types of social behaviors. In addition, the social sciences can help neuroscientists in becoming more aware of the cultural and political biases that they may implicitly display in their work. In recent years, neuroscientists have started to engage with such controversial topics as crime prevention (Aharoni et al., 2013), the pros and cons of technology (Levitin, 2014), and conflict resolution (Bruneau et al., 2012). In doing so, neuroscientists will have to grapple with the influence that their own social and political biases can have on their research. In setting out which ideological biases are likely to emerge, and in calling attention to the influence of these biases on academic research, the social sciences can provide partial relief (Whitehead, 2012).

It stands to reason to expect that the chances of enriching neuroscience with social theories are increased, when these efforts involve social science approaches consistent with the insights that have accumulated in neuroscience during the last decades. In the remainder of this paper, we focus on the second contribution that social theory has to offer brain research—serving as a source of inspiration for neuroscientific models. We do not do so by describing, in generic terms, what this contribution may entail. Instead, we believe that it is more helpful to demonstrate, with the help of a concrete example, how a particular social theory can be used to improve a prominent, though not undisputed, neuroscientific hypothesis. In this case, we concentrate on the somatic marker hypothesis, and show how it can be complemented by plural rationality theory. After describing the hypothesis itself, we discuss various conceptual and empirical criticisms it has received. We then argue that plural rationality theory can be used to specify the proposed somatic marker mechanism so as to meet some of these criticisms.

### The somatic marker hypothesis

The somatic marker hypothesis (Damasio, 1994, 1999, 2003) is currently one of the most widely discussed models of human decision-making in affective and social neuroscience. It incorporates, but also goes beyond, the key insights from these fields that we presented earlier. According to this hypothesis, somatic (i.e., bodily) states play a central role in decision-making processes. Becoming associated with a particular type of situation, they limit the scope of possible decisions that an individual takes into consideration, and bias the person toward choices that reflect previous responses made in similar situations. In reflecting accumulated experience, somatic markers provide a powerful, initial step in any decision-making process, bypassing the need to laboriously re-appraise all past responses to situations of a similar type and assessing all possible alternatives.

Let us consider this process in more detail (Damasio, 1996; Damasio and Carvalho, 2013). Throughout life, we build associations between categories of objects and events in the world on the one hand, and emotions and feelings on the other. In this view, emotions consist of bodily states that are triggered in response to “emotionally competent stimuli,” and the neuronal mapping of these states. Alterations in bodily state may include changes in the internal chemical milieu and visceral structures (such as heart rate, endocrine release, and smooth muscle contraction), as well as changes in the musculoskeletal system associated with approach or withdrawal behaviors. Central representations of body state are constantly updated through the brainstem and cortical nuclei, and somatosensing areas in the cortex such as the insula, SI, and SII. Mapped by the brain, these bodily changes may give rise to conscious sensation, perceived as feelings. Damasio and colleagues have proposed that the ventromedial prefrontal cortex (VMPFC) plays a pivotal role in coupling categories of events with their associated somatic markers, and the feelings that have arisen from them in the past. Whether acting unconsciously or consciously, somatic markers can bias decision-making in response to emotionally competent stimuli. With the help of neurotransmitter systems in the brain stem (including dopamine and serotonin), the activity of neurons subserving behavior and cognition within the cortex can be influenced.

There are two mechanisms by which somatic states can act. When a person is confronted with a particular event or object (a “primary inducer”), there is initially a process of appraisal and categorization. This can occur consciously, utilizing early sensory and higher-order association cortices, or subconsciously, via an evolutionarily older, subcortical route involving the thalamus. Once the primary inducer has been associated with a particular pleasurable or aversive emotional state, the amygdala will trigger bodily (somatic) changes characteristic of that state. These changes are evoked via a variety of brain structures that include sectors of the basal ganglia and basal forebrain, midbrain structures such as the ventral tegmental area, hypothalamus, and brainstem nuclei, including the periacqueductal gray. The resulting somatic states are then relayed back to the brain, where neuronal maps representing the state of the body are formed. Brain structures critical for this process include a variety of brainstem nuclei, and somatosensing areas in the cortex such as the insula, SI, and SII. When the mapping of these somatic changes is strong enough to reach consciousness, as is often the case, then feelings result as well. Finally, the mapping of evoked somatic states can bias activity in the dorsolateral prefrontal cortex, which plays a crucial role in reasoning and decision-making before an action or response is performed.

Of course, decision-making does not always involve direct experience of an actual event or object. Many decisions are made based on remembering or imagining a primary inducer. Damasio and colleagues propose that such “secondary inducers” can operate in two ways through the VMPFC. In the first, the somatic states characteristic of the corresponding primary inducer are triggered within the body—the “body loop” as described earlier—albeit in a weaker form. In the second—labeled the “as-if body loop”—the body is bypassed, and the amygdala acts directly in concert with those “mapping” brain structures in order to simulate a body state, as if they were being enacted in the body itself. In both mechanisms, the brain mapping of somatic states that would normally correspond to the presence of a primary inducer allows the probable bodily consequences of possible future action to be tested in advance. In this way, the brain-based image of body states can contribute to the planning of future behaviors.

In any given situation, many evaluative processes may be active. It is therefore hypothesized that a “winner takes all” process determines which overall somatic state results. Whether body states are triggered through the appraisal of primary or secondary inducers, the feedback signals generated by the body modulate activity in those brain structures supporting the initiation of somatic states in the first place. By modulating the threshold above which the neural components of the somatic marker mechanism become active, the induction of subsequent somatic states in a given situation can be facilitated or hindered.

Thus far, the somatic marker hypothesis has been supported by evidence from studies using lesion, experimental, neuroimaging, and psychopharmacological data (Reimann and Bechara, 2010; Cui et al., 2013). Nevertheless, the model has not escaped controversy. Three sets of criticisms have been leveled. First, the experimental data supporting the hypothesis have been challenged (Maia and McClelland, 2004; Dunn et al., 2006; Steingroever et al., 2013). This type of data has been collected with the help of the Iowa Gambling Task, a game in which players have to select cards from several decks, and in the process develop an emotional preference for the most advantageous deck. The empirical evidence thus gathered has been challenged on the grounds that: participants may be able to cognitively (rather than emotionally) determine which deck offers the highest earnings; it is unclear whether emotions follow or precede choices; the data from these experiments may also support alternative explanations; and significant variation exists among the strategies of healthy participants. In response, it has been asserted that none of these challenges are truly fatal to the somatic marker hypothesis, and that the hypothesis has also been supported by other types of data (Bechara et al., 2005; Guillaume et al., 2009; Turnbull et al., 2014). Moreover, these criticisms appear to assume that somatic markers are necessarily unconscious and/or replace reasoning. Yet, according to Damasio's hypothesis, somatic markers can be conscious as well as unconscious, and support (rather than replace) reasoning. A second critique states that the somatic marker model is not incorrect, but is incomplete. The model illuminates how body representations in the brain are central components of people's emotions and feelings toward objects and events. But it does not address why an individual experiences certain body representations (and emotions and feelings) rather than others when confronted with a particular object or event (Craig, 2002; Panksepp, 2003). As such, the model needs to be complemented. Both critiques boil down to a third claim, namely that it is important to further develop and specify the somatic marker hypothesis so as to allow additional testing (Ohira, 2010; Bartol and Linquist, 2015). It has for instance been suggested that alternative somatic markers may be at work for different types of decisions and situations (Bechara et al., 2005). Plural rationality theory can be of assistance in thus developing the model. The approach applies in particular to decision-making in highly complex and uncertain—so-called “ill-structured” (Simon, 1973)—social situations (Wildavsky, 1987). It can be used to further specify how the somatic marker mechanism works in this type of social situations.

### Using plural rationality theory to complement the somatic marker hypothesis

Plural rationality theory posits that a person evaluates a complex social situation by automatically (and often subconsciously) noting: (1) the amount of stratification and collectivity present within that situation; and then (2) comparing these scores to his or her preferred levels of stratification and collectivity for that type of situation (as based on previous experiences within that type of situation). If both these scores match, then a person will be favorably predisposed toward that situation. If not, then the person will tend to feel negatively about the situation.

This makes sense from a neuroscientific perspective. From that vantage point, we can ask: what are the characteristics that available features of social situations should exhibit in order to be useful in the evaluation of those social situations? First, in order to serve their role in the somatic marker mechanism, these elements would have to be present in each and every social situation. Second, this also means that people would have to able to easily and quickly display the types of social behavior that constitute these elements. Third, these aspects of social situations should be instantly recognizable for people, even at a young age. Fourth, people would be automatically and continuously scanning their social environments for these elements. Last, given the proposal that the emotional apparatus involved has early evolutionary origins, the types of behavior that make up these elements of social situations should be recognizable within other species as well, and in particular among those sitting closest to us on our evolutionary branch.

Grid (or stratification) and group (or collectivity) fulfill all these criteria. They are universal aspects of social situations. When people interact, they will behave according to perceived ranking differences and the degree to which they feel others are caring (Todorov et al., 2008). Indeed, these types of behavior may have a hormonal basis. It has been hypothesized that oxytocin promotes social approach behavior (high group), while arginine-vasopressin induces social withdrawal (low group) (Harari-Dahan and Bernstein, 2014). Moreover, there is some evidence that testosterone enables social dominance (grid) (van Honk et al., 2014). In addition, it has been argued that levels of stratification and collectivity are quickly perceived by the human brain due to the embodied nature of these forms of interacting (Fiske et al., 2009). Stratification and collectivity are typically accompanied by, associated with, and therefore easily re-presented through, certain bodily postures. Stratification is closely linked to vertical differences in body posture: those in charge are often placed higher up and look down on others, are bowed to, stretch their arms in victory, *etc* (Schubert, 2005). Collectivity is often experienced through physical closeness, such as shaking hands, standing shoulder to shoulder, embracing one another, grooming, and so on (Schubert et al., 2008). In a series of experiments, Thomsen and colleagues (Thomsen et al., 2011) have shown that 10–13 months old infants already rely on the relative size of agents to predict the outcome of dominance contests among them. This work has been interpreted as suggesting that, from a very early age, whenever a person observes a social situation, he or she first and foremost notices those physical features (for instance related to movement or posture) that are themselves directly embodied in the observer, i.e., that can trigger changes in the observer's body state^1^. Furthermore, when the human brain is in its “default state” (i.e., not engaged actively in a task), the social environment is still being scanned for human interactions in general, and stratification and collectivity in particular (Iacoboni et al., 2004). Last, both grid and group appear omnipresent in the social relations of other animals as well (Chiao, 2010; Swedell, 2012). In particular, de Waal's studies of chimpanzees and bonobos—the animal species with the most similar genetic make-up as humans—reveal patterns of interaction that come close to those distinguished in human behavior by Douglas (de Waal and Lanting, 1998; de Waal, 2007).

Different levels of stratification and collectivity are therefore excellent candidates for core categories with which humans evaluate social situations. Indeed, as we noted above, these have already attracted ample attention in brain research and social psychology. Plural rationality theory provides a comprehensive framework of human decision-making in complex social situations that is based on this hypothesis. Moreover, an abundance of empirical evidence in favor of plural rationality theory has been collected in social anthropology, sociology, and political science (Douglas, 1982; Verweij and Thompson, 2006; Olli, 2012). Plural rationality theory can therefore complement, and further specify, the somatic marker mechanism. Doing so results in the following testable model.

As described by the somatic marker hypothesis, whenever a person observes a social situation (or an opinion stated about such a situation), then brain processes related to the integration, abstraction and categorization of this scene will take place in the relevant sensory and association cortices. We propose that during this phase, the brain will be particularly attentive to the levels of grid and group that appear present in the social situation, or that appear to be promoted (or discouraged) by the opinion stated. This information about the levels of grid and group will then be passed on to either the amygdala (in case of direct experiences) or the VMPFC (in case of imagined or remembered social situations), through which their associated, characteristic somatic states will be either induced or simulated, respectively. We propose as well that when the initial process of categorization is completed, and a social situation (or an opinion thereof) has been recognized as belonging (or referring) to a particular category of situations, then this should also lead to the recall of the levels of stratification and collectivity preferred for this type of situation by the person in question. A comparison between actual and preferred levels of stratification and collectivity will take place, altering subsequent behavior in response to the social situation being experienced^2^.

Thus, plural rationality theory can be used to elaborate how the somatic marker mechanism may function in highly complex social situations. The call for such testable specifications has often been heard among the criticisms leveled at the somatic marker hypothesis. Complementing the hypothesis with the help of Douglas' plural rationality theory therefore illustrates how social science approaches can sometimes serve to strengthen neuroscientific concepts and models.

### Clearing up a conceptual conundrum confounding plural rationality theory

If empirically valid, then our proposal might even resolve a conundrum that has long worried plural rationality theorists (Thompson, 1982). Douglas (1978) derived four alternative ways of organizing (and supporting ways of viewing and experiencing nature, human nature, time, space, risk, *etc*.) by assigning high and low values to two underlying social dimensions. That is to say, grid and group are treated as dichotomous. However, they appear to be defined as continuous dimensions (namely, as variable levels of stratification and collectivity). Our model shows a way out of this conundrum. The strength of Douglas' proposal is the way in which a huge diversity in the organization and perception of social relations can arise through two simple, readily available, and highly informative features: grid and group. Both the categorization of complex social relations in terms of these two features, and the intimate relationship between these features and body states, earmark them as highly suitable candidates for the operation of the somatic marker mechanism, at least in highly complex social situations. Given that neuronal networks routinely function with the help of a threshold mechanism, it is plausible to expect that the grid and group dimensions, as part of the somatic marker mechanism, also operate on the basis of a threshold. If the threshold is met, then high grid or group is detected. If not, then low grid or group is detected. Moreover, once high or low levels of grid and group have been detected in a specific social situation, then (according to our proposal) these will be compared to preferred degrees of stratification and collectivity for that type of situation. Depending on the outcome of this comparison, the somatosensory pattern postulated by the somatic marker mechanism marks the specific situation as either pleasant or unpleasant, i.e., either appetitive or aversive emotions and feelings result. This would turn continuous variables into dichotomous ones, and thus justify the high/low distinction proposed by Douglas.

Although a division of grid and group into high or low—giving rise to the four categories of social life—might appear limited, for various reasons this framework is able to generate wide flexibility and diversity in the assessment of social relations. First, the classification of a given social scenario is not an all-or-nothing, one-off event. It arises over time in response to many factors indicative of social relations such as tone of voice, body language, visual appearance, *etc*. In line with plural rationality theory (Gross and Rayner, 1985; Olli, 2012), we suggest that the classification of social relations will occur at many points over the course of this emerging dynamic, resulting in a continually updated and re-calculated “average” classification. Each social scenario can differ in both the extent to which the four ways of organizing are represented, and the number of momentary classifications falling into each category. Therefore, rather than introducing an additional continuous variable into our model that represents the intensity of hierarchal, egalitarian, fatalist and individualist categories—or for that matter replacing the high/low division within grid and group itself with a continuous variable—a measure for their intensity emerges automatically over the course of real-life interactions. The more classifications that fall into one of the four categories of social relations, the more that category biases the overall classification and experience of the social scenario. This approach seems conceptually well suited to the simplicity of the somatic marker mechanism. Second, we propose that the threshold between high and low calculations for grid and group would itself be subject to modulation, thus introducing subject-specific variation in the classification of features and events into the four categories of social relations. If, for example, the grid threshold were to decrease for a given subject, then many more features and events would be defined as being “high” grid (i.e., being above threshold), biasing overall classifications in the hierarchical and fatalist categories. These two components of the classification process (the threshold at which a classification occurs, and its calculated intensity), coupled with a flexibility in their triggering of body states, result in a system for the processing of social relations that has a great subtlety, nuance, and individuality.

### Plural rationality theory and neuroimaging: experimental avenues

It will be useful to map, experimentally, the neuronal networks that support the detection, comparison, and evaluation of stratification and collectivity in social situations. We are planning two functional neuroimaging experiments that simultaneously investigate all four elementary forms of social relations distinguished in plural rationality theory. The first of these will expose participants to previously recorded visual media scenes depicting social interactions that can be characterized as individualistic, hierarchical, egalitarian, or fatalistic. Participants, numbering around 20, will be drawn from students previously trained and tested in recognizing plural rationality theory's categories. In the scanner, they will be asked to indicate for each scene whether the interaction can be categorized as individualistic, hierarchical, egalitarian, or fatalistic. Multivariate pattern analysis of brain activity for hierarchical (high grid/high group) and fatalistic (high grid/low group) scenes should then reveal brain responses that mediate high grid. Analysis of individualistic (low grid/low group) and egalitarian (low grid/high group) situations should uncover brain responses that mediate low grid. In this way, the neuronal correlates of high group (comparing egalitarian and hierarchical scenes) and low group (comparing individualistic and fatalistic situations) could also be revealed. A simple control condition would involve the observation and, later, classification of simple, performed motor tasks. In controlling for both sensory (visual and auditory) processing, as well as rudimentary decision making processes related to the classification of previously observed situations, contrasts between control and test conditions would reveal the machinery that underlies the processing and categorization of social relations in particular.

This first study investigates the conscious reflection on, and identification of, the four types of social relations arising in scenarios in which the subject plays no role. It will be useful to compare its results with those of a second study that concentrates on decision-making by participants while they are actively immersed in more “natural” social situations, in which the classification of social relations arises automatically, and does not follow from a conscious decision to do so. This second design will make use of priming techniques. One of the techniques available is the construction of alternative virtual social realities in which participants interact with anthropomorphic avatars (Przyrembel et al., 2012). We will build individualistic, egalitarian, fatalistic, and hierarchical virtual social realities. Participation in these virtual reality scenarios will prime subjects to think, feel, and act according to a certain set of social relations. After this priming component, participants will be confronted in the scanner with an image of one of the avatars, and tasked with making predictions (from a number of possible options) concerning the behavior of that avatar in a variety of social situations. By asking participants to engage with different virtual social realities, within-subject comparisons will become possible. As before, participants will number no more than 20, and multivariate pattern classification methods will be used to identify the neuronal correlates of grid and group. A control condition would again consist of observing and naming simple motor task.

These are only two examples of how our proposal for an extended somatic marker mechanism can be put to the test^3^. Other designs are surely possible, and need to be developed. Only a combination of designs, each with their own particular experimental strengths, will be able to shed light on the empirical validity of our model.

## Conclusion

We have argued that affective and social neuroscience need social theory, and *vice versa*. We have also shown how these processes of mutual support can be organized. The insights into cognition, emotion, decision-making, and social behavior that have been carefully built up and tested in neuroscience over the last few decades could provide an additional assessment of social theories. It would be less than ideal if such theories rested on assumptions and hypotheses about human cognition, emotion, and behavior that were incompatible with these insights. Yet, this appears to be the case for three leading approaches in the social sciences, namely rational choice theory, behavioral economics, and public policy, and post-structuralism. In contrast, Douglas' plural rationality theory is consistent with present-day affective and social neuroscience. As a consequence, this approach and similar ones (such as Fiske's relational models theory) may be of great use to the further development of neuroscience. They can offer conceptually sophisticated, and empirically supported, definitions of the social phenomena that neuroscience has become interested in exploring. They allow neuroscientists wading into public debates an opportunity to become more aware of the ideological assumptions lurking behind their research. Last, such theories can lead to suggestions as to the content of the models with which neuroscientists seek to explain how the human brain processes and evaluates social and political phenomena. In this article, we have for example shown how Douglas' plural rationality theory can be used to propose a more comprehensive and specific version of Damasio's somatic marker hypothesis. Achieving all this will not be easy, as it will require a mutually respectful interaction between different academic cultures. But it can be done, or so we feel.

### Conflict of interest statement

The authors declare that the research was conducted in the absence of any commercial or financial relationships that could be construed as a potential conflict of interest.

## Footnotes

^1^These processes of embodied cognition can be expected to involve mirror neurons, as these cells are active both when undertaking and observing a particular action (Gallese, 2005). Interestingly, Damasio and collaborators have argued that mirror neuron networks constitute part of the as-if body loop of the somatic marker mechanism (Damasio and Meyer, 2008; Immordino-Yang, 2008; Damasio, 2010).

^2^For simplicity's sake, we are leaving out the formation of conscious beliefs.

^3^Another avenue that we are considering is to expose subjects to real-world social scenarios with other human agents. Unknown to the test subjects, they would be interacting with actors playing carefully scripted roles designed to shape and bias their interactions toward one of the four different forms of social organization. (A company of stage actors in Berlin, called *Institut für künstlerische Forschung*, specializes in helping psychologists and neuroscientists create a certain social setting within the lab). Later during scanning, subjects would, as described earlier, be asked to make predictions about the behavior of those they have encountered in different social situations.
